# Efficient near-infrared up-conversion photoluminescence in carbon nanotubes

**DOI:** 10.1038/ncomms9920

**Published:** 2015-11-16

**Authors:** Naoto Akizuki, Shun Aota, Shinichiro Mouri, Kazunari Matsuda, Yuhei Miyauchi

**Affiliations:** 1 Institute of Advanced Energy, Kyoto University, Uji, Kyoto 611-0011, Japan; 2 PRESTO, Japan Science and Technology Agency, 4-1-8 Honcho Kawaguchi, Saitama 332-0012, Japan; 3 Graduate School of Science, Nagoya University, Chikusa, Nagoya 464-8602, Japan

## Abstract

Photoluminescence phenomena normally obey Stokes' law of luminescence according to which the emitted photon energy is typically lower than its excitation counterparts. Here we show that carbon nanotubes break this rule under one-photon excitation conditions. We found that the carbon nanotubes exhibit efficient near-infrared photoluminescence upon photoexcitation even at an energy lying >100–200 meV below that of the emission at room temperature. This apparently anomalous phenomenon is attributed to efficient one-phonon-assisted up-conversion processes resulting from unique excited-state dynamics emerging in an individual carbon nanotube with accidentally or intentionally embedded localized states. These findings may open new doors for energy harvesting, optoelectronics and deep-tissue photoluminescence imaging in the near-infrared optical range.

## Abstract (web summary)

Photoluminescent objects absorb light and then relax by emitting photons, usually with a lower energy. Here, the authors show that carbon nanotubes also emit larger energy photons thanks to one-phonon-assisted up-conversion, suggesting that nanotubes could be used as near-infrared up-converters.

## Graphical abstract

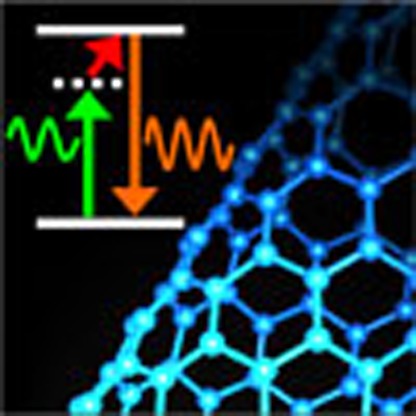

Photoluminescence phenomena normally obey Stokes' law of luminescence^1^, which states that the emission light wavelength (energy) is typically longer (lower) than its excitation counterpart. Up-conversion (or anti-Stokes) photoluminescence^2^ violates the Stokes' law of luminescence owing to additional energy gain induced by multiple photon^2^,^3^,^4^,^5^ or thermal (phonon) energy^6^,^7^ absorption. This unique photon up-conversion phenomenon only occurs efficiently in certain materials such as rare-earth-doped materials^2^,^6^,^8^ and some organic dyes^3^,^4^ by virtue of their specific electronic structures. Its potential usefulness in various applications such as photovoltaic energy conversion^3^,^9^, photoluminescence bio-imaging^8^,^10^,^11^,^12^,^13^, displays^14^, lasers^15^ and optical refrigeration of solids^6^,^16^ has attracted growing interest in recent years. However, especially in the near-infrared optical range, limitations in materials and combinations of excitation and emission photon energies available for efficient photon up-conversion have reduced its compatibility with a wide range of applications.

Single-walled carbon nanotubes (hereafter referred to as carbon nanotubes) consist of graphene sheets rolled up into quasi-one-dimensional tubular structures with diameters of the order of 1 nm (ref. 17). Depending on the rolling-up direction and their diameter defined by two indices (*n*, *m*)^18^, nanotubes act as direct gap semiconductors with band gaps of the order of 1 eV and emit photoluminescence in the near-infrared optical range^19^ because of recombination of excitons (electron and hole pairs bound via attractive Coulomb interactions)^20^,^21^,^22^. Since the first observation of efficient Stokes photoluminescence from individual carbon nanotubes^19^,^23^, numerous distinctive luminescence properties of nanotubes and their applications have been uncovered over the past decade^24^,^25^,^26^,^27^. However, this report reveals an additional remarkable characteristic of carbon nanotubes as photoluminescent materials.

Here, we demonstrate that carbon nanotubes^17^ are new up-conversion phosphors showing efficient near-infrared to near-infrared up-conversion photoluminescence at room temperature with an energy gain of up to a few hundreds of milli-electronvolts, corresponding to light wavelength shortening of a few hundreds of nanometres. This photon up-conversion does not originate from common coherent two-photon absorption or anti-Stokes Raman processes. The observed phenomena are attributed to efficient excitonic up-conversion processes gaining energy by absorbing high-energy (∼100–200 meV) optical phonons characteristic in carbon nanotubes, although the phonon energy much exceeds the ambient thermal energy (only ∼26 meV) and corresponding phonon number available for the up-conversion is considerably small. This apparently anomalous process stems from unique excited-state dynamics emerging in an individual carbon nanotube with accidentally or intentionally embedded localized states. These findings may lead to new opportunities for near-infrared and thermal energy harvesting, optoelectronics and deep-tissue up-conversion photoluminescence imaging using conventional silicon-based image sensors and low-cost near-infrared light sources.

## Results

### Optical imaging of the up-conversion light emission

**Figure 1:**
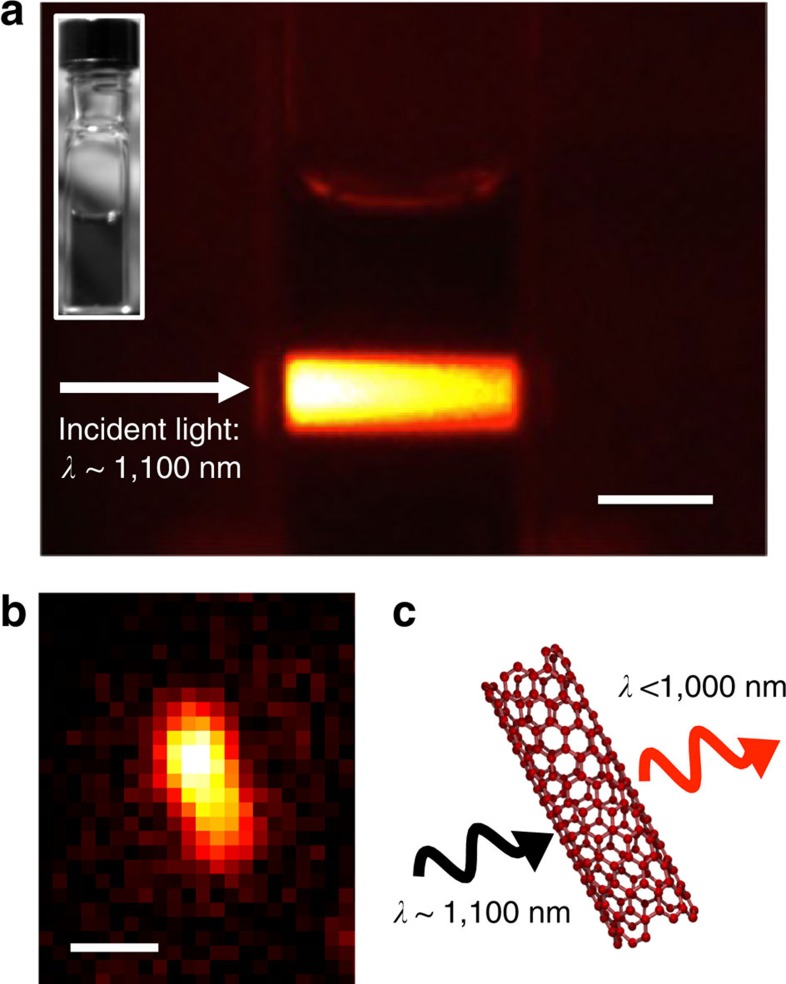
Up-conversion light emission of carbon nanotubes. (**a**) Optical image showing light emission from (6,5)-rich carbon nanotubes dispersed in D_2_O using surfactant (sodium dodecyl benzene sulfonate, Methods section) excited by incident light of wavelength ∼1,100 nm (selected wavelength range of 1,075–1,125 nm, power density=200 mW cm^−2^, collimated). The photoemission image was selectively detected in the 950–1,000 nm wavelength range using optical filters. The micelle-suspended nanotube dispersion was placed in a quartz cell (inset). Scale bar, 5 mm. (**b**) Microscopic image of the up-conversion light emission from an individual nanotube taken under the same excitation wavelength (∼1,100 nm) used in the experiment shown in **a**. Approximate excitation power density was 4 kW cm^−2^ (Methods section). Scale bar, 1 μm. (**c**) Optical processes observed in the experiment shown in **a** and **b**. A shorter wavelength (higher energy) photoemission (<1,000 nm) results from excitation at longer wavelength (lower energy) incident light (∼1,100 nm).

Figure 1a shows an optical image of photoemission from (6,5)-rich carbon nanotubes dispersed in D_2_O (Methods section) in a standard quartz cell. The incident photon wavelength (energy) was set to ca. 1,100 nm (1.13 eV) and the photoemission image ranging between 950 and 1,000 nm (1.24–1.31 eV) was selectively captured using optical filters. The power density of the collimated incident light equalled 200 mW cm^−2^, which was too weak to induce coherent two-photon excitation photoluminescence^21^,^22^. Although the incident photon energy (wavelength) was considerably lower (longer) than the detected photon energy (wavelength), photoemission was clearly observed along the incident light path. We also confirmed that this light emission is from individual carbon nanotubes by directly observing microscopic light emission image of an individual nanotube under the up-conversion excitation condition as shown in Fig. 1b. This situation is schematized in Fig. 1c.

### Up-conversion photoemission spectra

**Figure 2:**
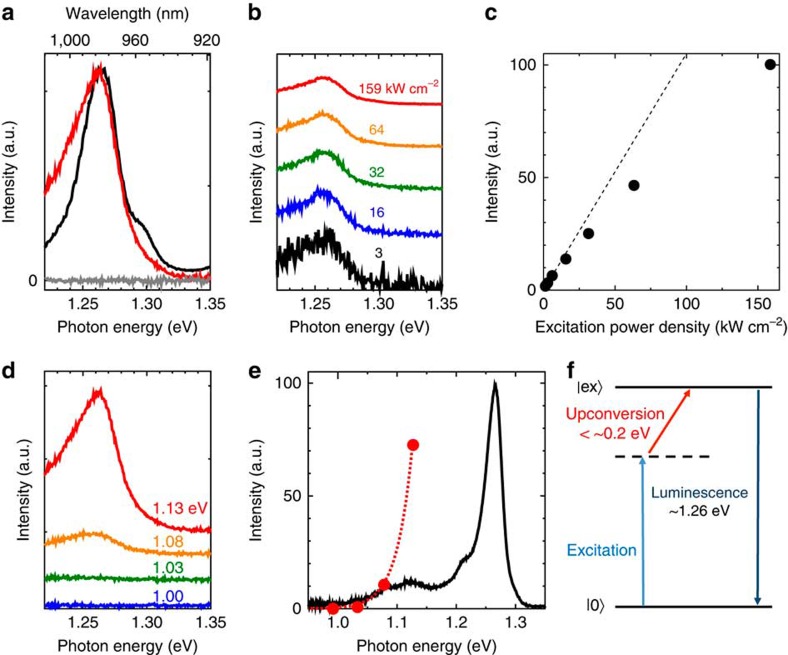
Excitation power and energy dependences of the up-conversion photoemission. (**a**) Photoemission spectra of carbon nanotubes excited at incident photon energies of 1.13 (1,100 nm, red curve) and 2.18 eV (568 nm, black curve). The gray curve is a spectrum taken for a carbon nanotube-free surfactant solution excited at 1.13 eV for comparison. The intensity was normalized so that the noise level appears to be comparable to the spectrum of carbon nanotubes excited at 1.13 eV (red curve). The excitation band width was 10 nm. (**b**) Excitation power density dependence of photoemission spectra excited at 1.13 eV. The spectra for excitation densities from 3 to 159 kW cm^−2^ are shown. Each spectrum is normalized by corresponding excitation power density. (**c**) Photoemission intensity (from data shown in **b**) plotted as a function of excitation power density. The dotted line corresponds to linear function for comparison. (**d**) Excitation photon energy dependence of the photoemission spectra. In **b** and **d**, each spectrum was vertically shifted for comparison, and corresponding excitation power densities (for **d**) or excitation photon energies (for **d**) are indicated on each spectrum for clarity. (**e**) Up-conversion photoemission intensity (red circles, from spectra in **d**) as a function of excitation photon energy, plotted together with normal photoluminescence spectrum at an excitation energy of 2.18 eV (black curve) for comparison. The dotted red curve is a guide to the eye. (**f**) Energy diagram for the observed photon up-conversion processes. Solid lines represent the ground (|0>) and free exciton (|ex>) states in carbon nanotubes. The dashed line corresponds to an intermediate state in the up-conversion process.

Figure 2a compares photoemission spectra of nanotubes excited with 1.13 (1,100 nm; red curve) and 2.18 eV (568 nm; black curve) photons, respectively. The grey curve shows control data obtained for a nanotube-free surfactant solution excited at 1.13 eV. The emission peak detected at ca. 1.26 eV upon excitation at 2.18 eV (resonant with the second sub-band exciton energy *E*_22_ of (6,5) nanotubes) corresponds to the photoluminescence induced by exciton recombination in the first sub-band (*E*_11_) of (6,5) nanotubes^23^. The photoemission spectrum obtained at an excitation energy of 1.13 eV exhibited a peak at ca. 1.26 eV. The similar peak energies for excitation energies of 1.13 and 2.18 eV indicate that the photoemission mainly originates from (6,5) nanotubes (Supplementary Note 1 and Supplementary Fig. 1).

### Excitation power dependence

The excitation power density dependence was examined to clarify the origin of the observed up-conversion photoemission. The spectral shape hardly changed with excitation power density variations (Fig. 2b). Figure 2c shows the up-conversion photoemission intensity as a function of excitation power density, revealing a weak sublinear dependence between these parameters. These observations exclude any possible photoemission induced by multiple-photon absorption (coherent or incoherent) or Auger recombination-mediated up-conversion^28^ as origins for the observed phenomena because these mechanisms usually display a superlinear (quadratic or more) excitation power dependence.

### Excitation photon energy dependence

Next, the excitation photon energy dependence of the up-conversion photoemission spectra was evaluated (Fig. 2d). The up-conversion intensity strongly depended on the excitation photon energy (wavelength) but spectral shape and peak positions remain nearly intact upon excitation photon energy variations. This observation rules out the possibility that the up-conversion photoemission results from anti-Stokes Raman scattering because Raman processes induce clear peak shifts according to excitation energy changes.

The exclusion of these potential processes suggests that the observed up-converted photoemission is attributable to photoluminescence induced by one-photon absorption followed by an internal excitonic up-conversion process in a carbon nanotube. Therefore, the observed phenomena are referred to as up-conversion photoluminescence in the remainder of the manuscript. Let us now consider the mechanism that governs this efficient excitonic up-conversion. The excitation photon energy dependence of the up-conversion intensity may provide a clue to the mechanism. Figure 2e compares the observed up-conversion photoluminescence intensity (red circles) as a function of excitation photon energy with a normal (Stokes) photoluminescence spectrum (black curve) obtained at an excitation energy of 2.18 eV. The threshold excitation photon energy required to achieve an observable up-conversion photoluminescence reaching ca. 1.3 eV amounted to 1.03–1.08 eV. A diagram showing excitation and emission photon energies for the observed up-conversion processes is shown in Fig. 2f.

### Temperature dependence

**Figure 3:**
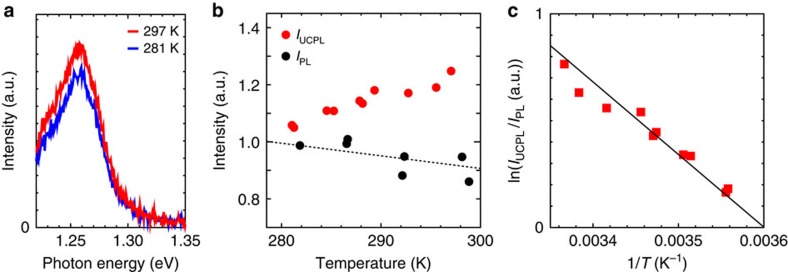
Temperature dependence of the up-conversion photoluminescence. (**a**) Up-conversion photoluminescence spectra at 297 (red curve) and 281 K (blue curve) for an excitation energy of 1.13 eV. (**b**) Up-conversion (red circles, excitation energy=1.13 eV, *I*_UCPL_) and normal photoluminescence intensities (black circles, excitation energy=2.18 eV, *I*_PL_). The temperature-dependent normal photoluminescence intensity is fitted with a linear function (dotted line), which was used to normalize the up-conversion photoluminescence intensity at each temperature in **c**. (**c**) Arrhenius plot of the up-conversion intensity (*I*_UCPL_) normalized by the normal photoluminescence intensity (*I*_PL_) at the corresponding temperature (red squares). *I*_UCPL_ was divided by *I*_PL_ to account for the temperature-dependent variation of *I*_PL_. The black solid line represents the Arrhenius equation for an activation energy of 120 meV.

The threshold energy gain close to the maximum optical phonon energy (∼0.2 eV) in carbon nanotubes^29^ and increasing up-conversion efficiency for smaller energy gain imply that phonon-assisted processes may induce internal up-conversion in carbon nanotubes. Therefore, temperature-dependent studies on the up-conversion photoluminescence were conducted. Figure 3a compares up-conversion photoluminescence intensities at 297 and 281 K at an excitation energy of 1.13 eV. The up-conversion photoluminescence intensity exhibited high sensitivity to small temperature variations and increased with increasing temperature. Figure 3b shows the up-conversion (red open circles) and normal photoluminescence intensities (black open circles) as functions of temperature. Up-conversion and normal photoluminescence intensities displayed opposite trends with respect to temperature (Fig. 3b). The up-conversion photoluminescence intensity increased, whereas its normal counterpart slightly decreased when the temperature increased.

Figure 3c shows an Arrhenius plot of the up-conversion intensity (*I*_UCPL_) divided by the normal photoluminescence intensity (*I*_PL_) at each corresponding temperature. The solid line in Fig. 3c represents the Arrhenius equation (∝exp(−*E*_a_/*k*_B_*T*) where *E*_a_, *k*_B_ and *T* are the activation energy, Boltzmann constant and temperature) for an *E*_a_ value of 120 meV. (See also Supplementary Fig. 2 for data obtained at an excitation energy of 1.08 eV). The experimental trend was well reproduced with an *E*_a_ value closely matching the typical energy gain (120–130 meV) at an excitation energy of 1.13 eV, strongly suggesting that the internal exciton up-conversion originates from phonon absorption processes in carbon nanotubes, rather than other possible processes such as triplet–triplet annihilation^3^,^30^. Note that the activation energy *E*_a_ nearly equal to the energy gain indicates one-step up-conversion via absorption of an optical phonon with much higher energy (∼100–200 meV) than *k*_B_*T* (∼26 meV), distinct from common thermal activation process in which the additional energy is mainly provided by absorption of multiple phonons with energy comparable to or lower than *k*_B_*T*.

### Intermediate states in up-conversion processes

Now that the up-conversion photoluminescence has been clearly attributed to phonon-assisted processes, the reason for the efficient phonon-assisted up-conversion with an energy gain reaching 5–10 *k*_B_*T* needs to be elucidated. The weak sublinear dependence of the up-conversion photoluminescence intensity on the excitation power density (Fig. 2c) may provide insight into this anomalous phenomenon. The sublinear dependence implies the existence of an intermediate state with finite lifetime causing state-filling-induced saturation effect. Because it lies at lower energy than the *E*_11_ free exciton state, this state may be a mid-gap localized state^31^,^32^,^33^,^34^ unintentionally generated in carbon nanotubes during sample preparation because some types of these states^32^,^33^,^34^,^35^,^36^ are optically active and can be photoexcited directly by near-infrared photons.

**Figure 4:**
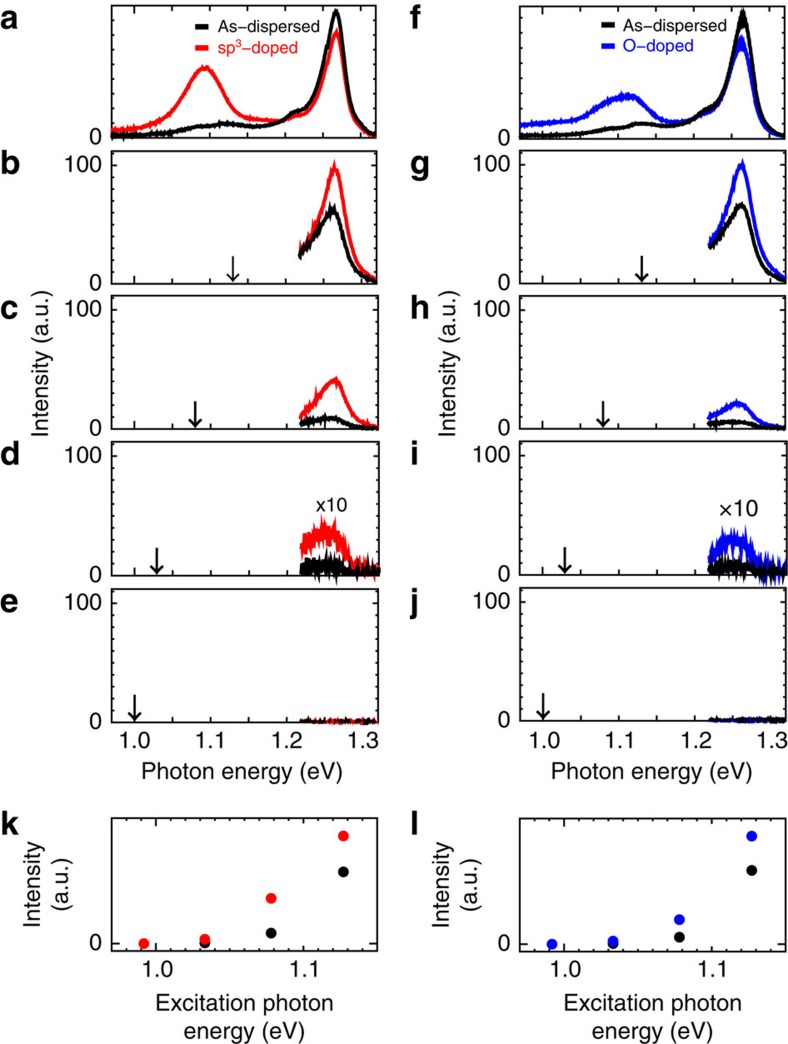
Up-conversion photoluminescence with intentionally doped states. (**a**,**f**) Photoluminescence spectra of as-dispersed (black curves) and 0D-doped carbon nanotubes (red and blue curves for (**a**) sp^3^-and (**f**) oxygen-doped structures, respectively). **a** and **f** show normal photoluminescence spectra of each sample excited at 2.18 eV. (**b**–**e**, **g**–**j**) Up-conversion photoluminescence spectra of samples excited at (**b**,**g**) 1.13, (**c**,**h**) 1.08, (**d**,**i**) 1.03 and (**e**,**j**) 1.00 eV, respectively. Vertical arrows in each panel indicate the corresponding excitation photon energy. Spectra in **d**,**i** for an excitation energy of 1.03 eV were magnified 10 times. (**k**,**l**) Excitation energy-dependent up-conversion photoluminescence intensities of as-dispersed (black circles) and 0D-doped (red and blue circles for sp^3^- and oxygen-doped structures, respectively) carbon nanotubes.

To test this hypothesis, localized states were intentionally embedded in carbon nanotubes by chemical treatments^32^,^33^,^34^, and up-conversion photoluminescence changes were examined. Carbon nanotubes with oxygen- and sp^3^-defect-derived optically active localized (zero-dimension (0D)-like) states were prepared according to procedures originally developed by Ghosh *et al.*^32^ and Piao *et al.*^34^ with some modifications in selections of chemicals and experimental parameters, respectively (Methods section). Figure 4a,f compare the normal photoluminescence spectra of as-dispersed (black curves) and sp^3^-defect (red curve) or oxygen-doped (blue curve; hereafter referred to as 0D-doped) carbon nanotubes. A relatively broad and intense photoluminescence band emerged around 1.1 eV after chemical treatment, whereas the original *E*_11_ photoluminescence intensities decreased only slightly for 0D-doped nanotubes. Figure 4b–e,g–j show up-conversion photoluminescence spectra of as-dispersed (black curves) and 0D-doped carbon nanotubes (coloured curves) excited at 1.13, 1.08, 1.03 and 1.00 eV, respectively. Note that the 0D-doped nanotubes displayed a considerably enhanced up-conversion photoluminescence intensity compared with their as-dispersed counterparts regardless of origins of the localized states.

Figure 4k,l compare the excitation energy-dependent up-conversion photoluminescence intensities of as-dispersed and 0D-doped nanotubes. The up-conversion photoluminescence intensity increased dramatically at the excitation energy of 1.08 eV for 0D-doped nanotubes. Because this energy closely matched the photoluminescence peak energy of the localized excitons (Fig. 4a,f), this enhancement is attributable to exciton up-conversion from the additional localized states. The temperature (Supplementary Fig. 3) and the excitation power density dependences (Supplementary Note 2 and Supplementary Fig. 4) of the up-conversion photoluminescence were also assessed for the 0D-doped nanotubes. The results were similar to those obtained for as-dispersed nanotubes. This strongly suggests that the embedded localized states act as intermediate states in up-conversion processes. Moreover, the strong enhancement of the up-conversion photoluminescence achieved by simple chemical treatments is attractive for their applications as up-conversion phosphors.

## Discussion

We deduced the up-conversion quantum yield of the localized excitons to the free excitons using the difference between up-conversion photoluminescence intensities before and after intentional localized state doping (see Supplementary Discussion and Supplementary Fig. 9 for details). It was of the order of 10^−1^ for an energy gain of 120 meV even at room temperature. This highly efficient phonon-assisted exciton up-conversion showing an energy gain much exceeding the thermal energy appears anomalous but may be understood as a consequence of rapid exciton migration along the nanotube axis^37^ as described below. The efficient exciton scattering by acoustic phonons^38^ or random extrinsic surface potentials^39^ that drive rapid diffusive exciton migration^37^,^40^,^41^,^42^,^43^,^44^ surpassing >100 nm along the nanotube axis spatially separates the up-converted excitons from their original localized state immediately after up-conversion and strongly reduces their probability of returning to this original state (see Supplementary Discussion and Supplementary Figs 10–12 for details). This unique exciton dynamics emerging in a carbon nanotube with embedded 0D-like localized states enables efficient phonon-assisted exciton up-conversion with an energy gain exceeding the thermal energy by one order of magnitude at room temperature. A potential enhancement of the exciton–phonon coupling resulting from exciton localization^34^,^45^,^46^ may also contribute to the efficient phonon-assisted exciton up-conversion in carbon nanotubes.

Finally, let us discuss the merits of the up-conversion excitation wavelength of the nanotubes lying in a specific near-infrared optical range (*λ*=1,000–1,400 nm). This wavelength range is known as ‘second biological transparency window' (NIR II), in which the light penetration depth into biological tissues is maximized, whereas photo-induced degradation and autofluorescence are minimized^25^,^47^. Indeed, carbon nanotubes are expected to serve as promising phosphors for NIR II deep-tissue imaging, and biocompatible and luminescent nanotube dispersion has already been developed^25^,^47^. A chief obstacle to this imaging approach has been the unsuitability of common silicon-based image sensors because of their vanishing sensitivity for NIR II photons. The up-conversion photoluminescence of the (6,5) carbon nanotubes with the inverted excitation (in the NIR II range) and emission (<1,000 nm, so-called NIR I range) wavelengths may solve this problem and facilitate deep-tissue bio-imaging by taking advantage of the large penetration depth of NIR II photons and common silicon-based image sensors (Supplementary Notes 3–5 and Supplementary Figs 5–8). Because up-conversion occurs as a one-photon process in carbon nanotubes, it does not require costly ultrafast high power lasers and high-density excitation conditions unlike conventional multi-photon excitation imaging. Furthermore, up-converted photocarriers in carbon nanotubes may be directly extracted as electronic current by configuring photovoltaic devices because of their quasi-one-dimensional structures. Therefore, these findings may also lead to new routes for optoelectronics and heat and photon harvesting below the optical gap in the near-infrared region.

## Methods

### Sample preparation

(6,5)-rich CoMoCAT carbon nanotubes (Southwest NanoTechnologies, USA) were dispersed in D_2_O in the presence of 0.2% (w/v) sodium dodecyl benzene sulfonate under moderate bath sonication for 60 min and vigorous sonication using a tip-type sonicator for 40 min before centrifugation at an acceleration of 130,000*g* for 4 h (ref. 19). For the experiments shown in Fig. 4, additional localized states were embedded in carbon nanotubes by atomic oxygen^32^ or sp^3^-defect doping^34^ that reduce the band gap and exciton energy locally. Oxygen- and the sp^3^-defect-doped carbon nanotubes were prepared according to procedures originally developed by Ghosh *et al.*^32^ and Piao *et al.*^34^, respectively, with different chemical and experimental parameter selections^33^,^48^. In brief, the isolated nanotube dispersion was combined with a small amount of D_2_O containing dissolved ozone and left under the illumination of a desk lamp (ca. 5 mW cm^−2^) overnight, allowing moderate atomic oxygen doping to generate sparse oxygen-derived local states. To achieve sp^3^-defect doping, the nanotube dispersion was combined with a small amount of D_2_O-containing dissolved organic 4-bromobenzenediazonium tetrafluoroborate salts^34^ and left in the dark for at least 10 days, allowing moderate sp^3^-defect doping in carbon nanotubes to generate sparse sp^3^-defect-derived localized electronic states. Optical absorption spectra of these samples are presented in Supplementary Fig. 1.

### Optical measurements

Optical measurements were conducted using a broadband supercontinuum white light source (Fianium, SC450) for excitation. Incident light wavelengths were selected using a monochromator for normal Stokes emission measurements and optical filters for up-conversion photoemission measurements. The typical excitation band width was 10 nm for spectroscopic measurements and any unwanted stray light was carefully removed using multiple optical filters. Normal photoluminescence measurements were performed using monochromated light at a wavelength of 568 nm (2.18 eV) that matches the *E*_22_ optical resonance in (6,5) carbon nanotubes for photoexcitation. The optical image showing up-conversion photoemission phenomena (Fig. 1) was taken using an electron multiplying charge coupled device (EMCCD) camera (Princeton Instruments, ProEM). The microscopic up-conversion photoemission imaging of an individual nanotube shown in Fig. 1b was performed using an inverted microscope and a 1.4 NA, × 100 objective under epi-illumination of the near-infrared incident light (1,075–1,125 nm, approximate power density of 4 kW cm^−2^). For the microscopic imaging, the individually dispersed nanotubes were immobilized using an agarose gel matrix^37^. During spectral measurements shown in Figs 2, 3, 4, the nanotube dispersion was set in a quartz cell with optical path of 1 mm. Incident light was focused on the sample in the quartz cell as a 4–6-μm spot using an achromatic lens, and light emitted from the samples was collected by an achromatic lens and transferred to a confocal optical set-up. The near-infrared photoemission was recorded using a liquid nitrogen cooled Si-CCD or an InGaAs array detector. Detection sensitivity variations were corrected using a standard light source (Ocean Optics, LS-1). The spectral resolution typically equalled 2 nm. We used spectral-integrated intensity for the analyses of the photoemission intensity as functions of excitation power density or temperature. The nanotube dispersion temperature was directly measured in a quartz cell using a dipped thin thermocouple. For the measurements of temperature dependence, nanotubes in the quartz cell were gradually cooled using iced water or allowed to warm up by natural convection. Optical spectra were acquired at temperatures ranging from 280 K to room temperature (ca. 300 K). The temperature typically varied by 1.5 K during each spectral measurement.

## Additional information

**How to cite this article:** Akizuki, N. *et al.* Efficient near-infrared up-conversion photoluminescence in carbon nanotubes. *Nat. Commun.* 6:8920 doi: 10.1038/ncomms9920 (2015).

## Supplementary Material

Supplementary Information Supplementary Figures 1-12, Supplementary Notes 1- 5, Supplementary Discussion and Supplementary References.
