# Supplementary material for: Efficient near-infrared up-conversion photoluminescence in carbon nanotubes
Source: Nat Commun. 2015 Nov 16;6:8920. doi: 10.1038/ncomms9920 (PMC4660356; doi:10.1038/ncomms9920)
Supplement: Supplementary Information — Supplementary Figures 1-12, Supplementary Notes 1- 5, Supplementary Discussion and Supplementary References. [file ncomms9920-s1.pdf]

Supplementary Figures

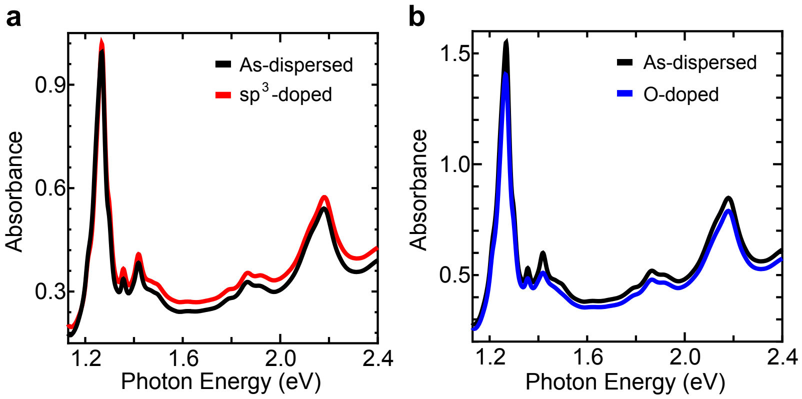

Supplementary Figure 1 | Optical absorption spectra of studied carbon nanotubes. (**a**,**b**) Optical absorption spectra of as-dispersed (black curves), (**a**) sp^3-doped (red curve), and (**b**) oxygen-doped (blue curve) (6,5)-rich carbon nanotubes.

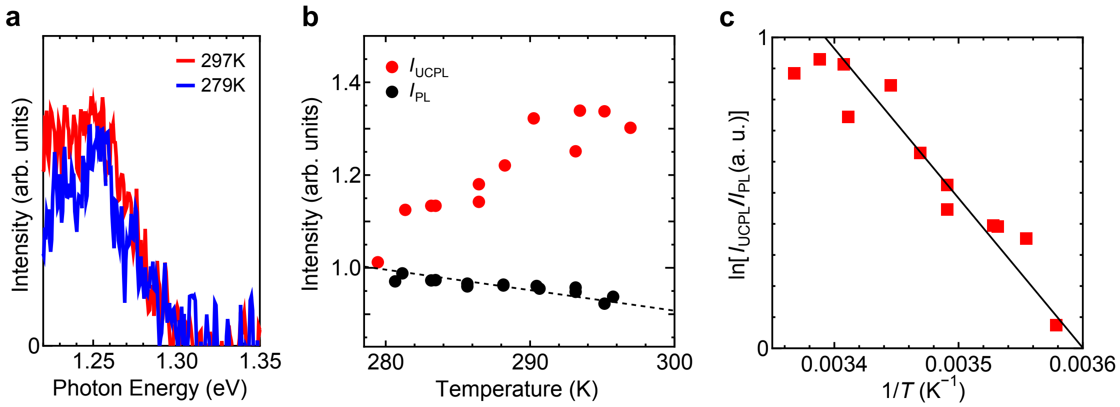

Supplementary Figure 2 | Temperature dependence of the upconversion photoluminescence intensity at an excitation energy of 1.08 eV. (**a**) Upconversion photoluminescence spectra at 297 (red curve) and 279 K (blue curve) obtained at an excitation energy of 1.08 eV. (**b**) Upconversion (red circles, excitation energy = 1.08 eV, I_{UCPL}) and normal photoluminescence intensities (black circles, excitation energy = 2.18 eV, I_{PL}). The temperature dependence of the normal photoluminescence intensity was fitted by a linear function (dotted line), which was used to normalize the upconversion photoluminescence intensity in **c**. (**c**) Arrhenius plot of the upconversion intensity normalized by the normal photoluminescence intensity at the corresponding temperature (red squares). The solid line corresponds to the Arrhenius equation for an activation energy of 170 meV.

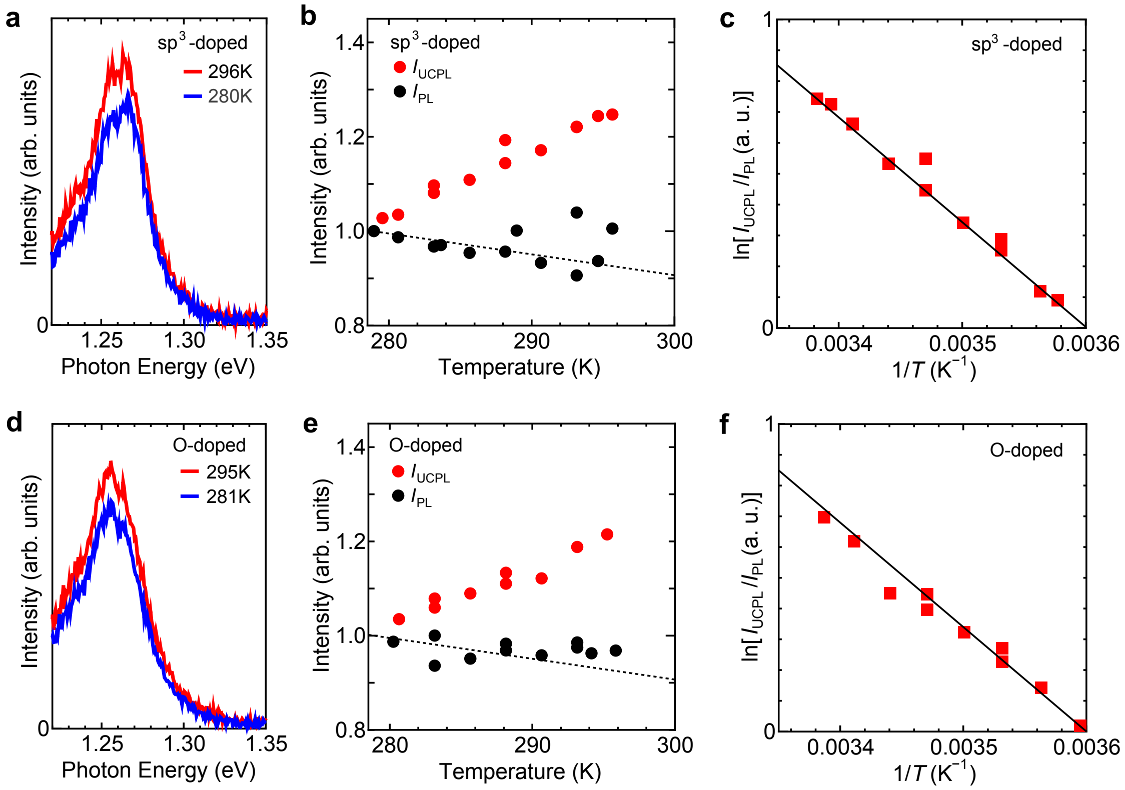

Supplementary Figure 3 | Temperature dependence of the upconversion photoluminescence of 0D-doped carbon nanotubes. Upconversion photoluminescence spectra obtained at an excitation photon energy of 1.13 eV and high (296 or 295 K, red curves) and low (280 or 281 K, blue curves) temperatures for (**a**) sp^3-doped and (**d**) oxygen-doped nanotubes. **b**,**e**, Upconversion (red circles, excitation energy = 1.13 eV, I_{UCPL}) and normal photoluminescence intensities (black circles, excitation energy = 2.18 eV, I_{PL}) as functions of temperature. The temperature dependence of I_{PL} was fitted by a linear function (dotted line), which was used to normalize I_{UCPL} in **c**,**f**. Arrhenius plots of I_{UCPL} normalized by I_{PL} at the corresponding temperature (red squares) for (**c**) sp^3-doped and (**f**) oxygen-doped carbon nanotubes. The solid lines correspond to the Arrhenius equation for an activation energy of 120 meV.

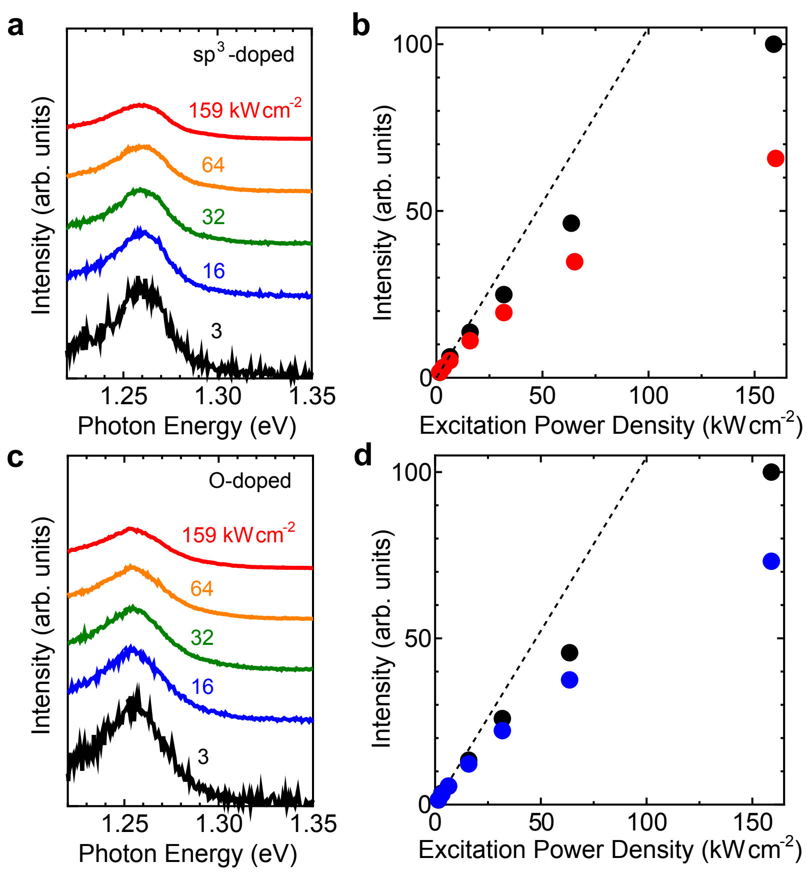

Supplementary Figure 4 | Excitation power density dependence of the upconversion photoluminescence of 0D-doped carbon nanotubes. Upconversion photoluminescence spectra for (**a**) sp^3-doped and (**c**) oxygen-doped carbon nanotubes measured under various excitation power densities. Upconversion photoluminescence intensities of (**b**) sp^3-doped (red circles) and (**d**) oxygen-doped (blue circles) carbon nanotubes from spectra shown in **a** and **c** as functions of excitation power density. Black circles represent data for as-dispersed carbon nanotubes. The dotted lines in **b** and **d** correspond to linear function for comparison.

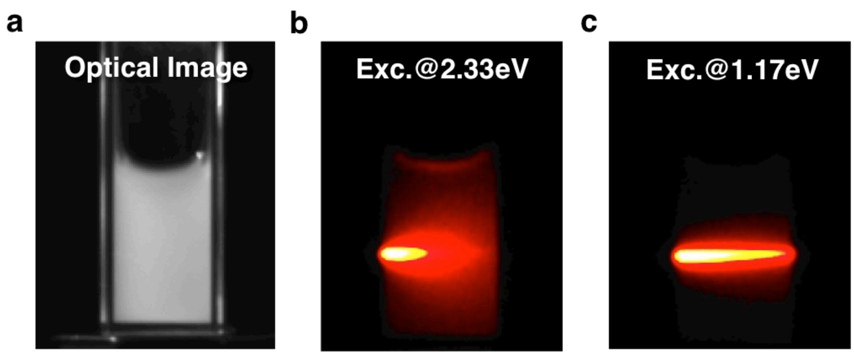

Supplementary Figure 5 | Comparison of light penetration depths into highly scattering media. (**a**) Normal optical image of 0.1% Intralipid® solution containing a small amount of carbon nanotubes in a standard quartz cell. (**b**,**c**) Photoluminescence images obtained at excitation photon energies of (**b**) 2.33 and (**c**) 1.17 eV. The excitation beams were collimated (beam diameter = 1 mm) and excitation power used for the measurements were (**b**) 1.4 and (**c**) 11 mW, respectively.

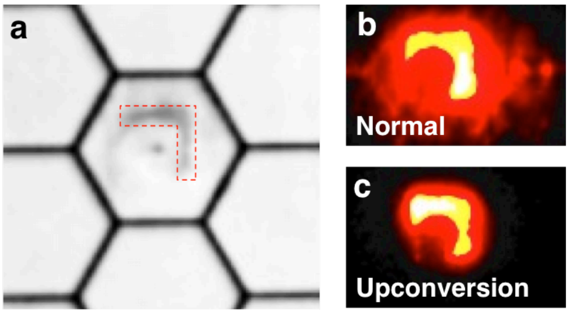

Supplementary Figure 6 | Comparison of normal and upconversion photoluminescence images. (**a**) Optical image of carbon nanotube dispersion sealed in transparent silicone elastomer held in a glass dish. Nanotube dispersion was held in the region indicated by red dashed lines. The sample was placed on a paper with a printed hexagonal grid (6mm each side). (**b,c**) Photoluminescence image of the sample under (**b**) normal (2.33 eV) and (**c**) upconversion (1.17 eV) excitation conditions. The approximate excitation power densities for normal and upconversion photoluminescence imaging were 0.4 and 6 $mWcm^{-2}$, respectively. These excitation densities gave almost same photoluminescence intensity for normal and upconversion excitation conditions.

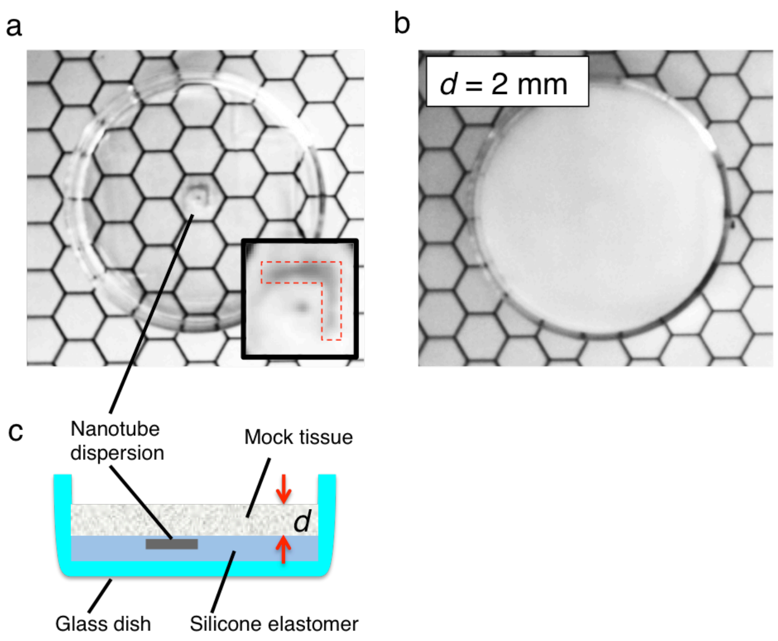

Supplementary Figure 7 | Carbon nanotubes placed behind a mock tissue. (**a**) Optical image of a glass dish containing carbon nanotube dispersion sealed in transparent silicone elastomer. Inset shows the magnified image of the sealed nanotube dispersion. Nanotube dispersion was held in the region indicated by red dashed lines. (**b**) Optical image of the same sample covered with the mock tissue (1% Intralipid® solution [1]) with thickness *d* = 2 mm. (**c**) Schematic representation of the sample cross-section covered with the mock tissue.

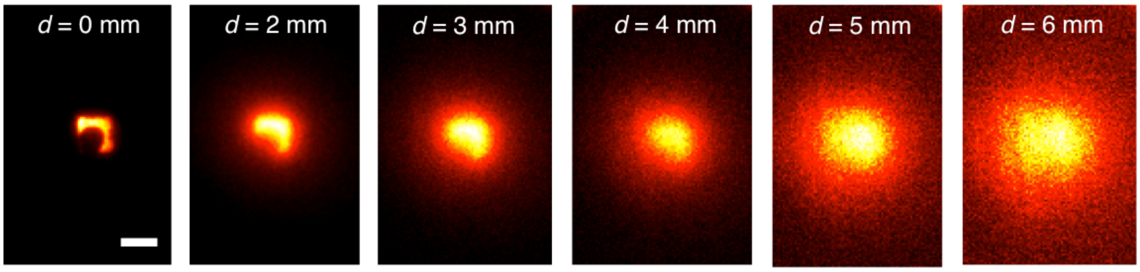

Supplementary Figure 8 | Upconversion photoluminescence images of a carbon nanotube dispersion held behind a mock tissue. The images for the tissue thickness *d* ranging from 0 (without mock tissue) to 6 mm are shown. The upconversion photoluminescence at wavelengths ranging from 950 to 1000 nm (1.24–1.31 eV) was selectively captured using optical filters. Images were acquired using a Si-based EMCCD camera. The total integration time amounted to 10 s for each image acquisition. The scale bar indicates 5 mm.

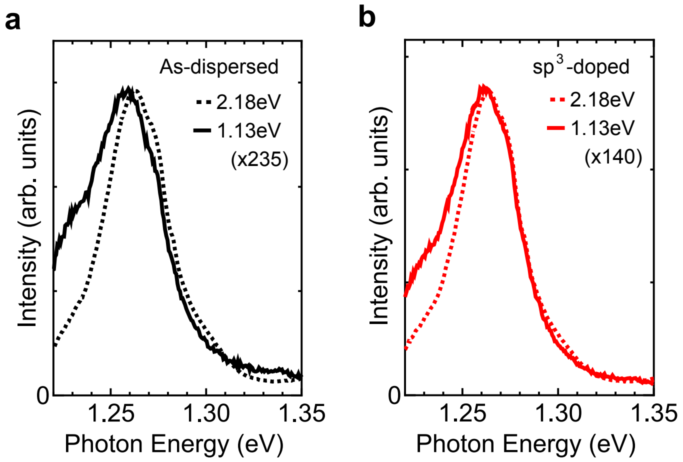

Supplementary Figure 9 | Normal and upconversion photoluminescence spectra of as-dispersed and sp^3-doped nanotubes. Normal (dotted curves, excitation energy = 2.18 eV) and upconversion (solid curves, excitation energy = 1.13 eV) photoluminescence spectra of (**a**) as-dispersed and (**b**) sp^3-doped carbon nanotubes. These spectra were acquired at a spectral resolution of 13 nm. Each spectrum was normalized using excitation power density and integration time. The upconversion spectra were magnified (**a**) 235 and (**b**) 140 times for comparison.

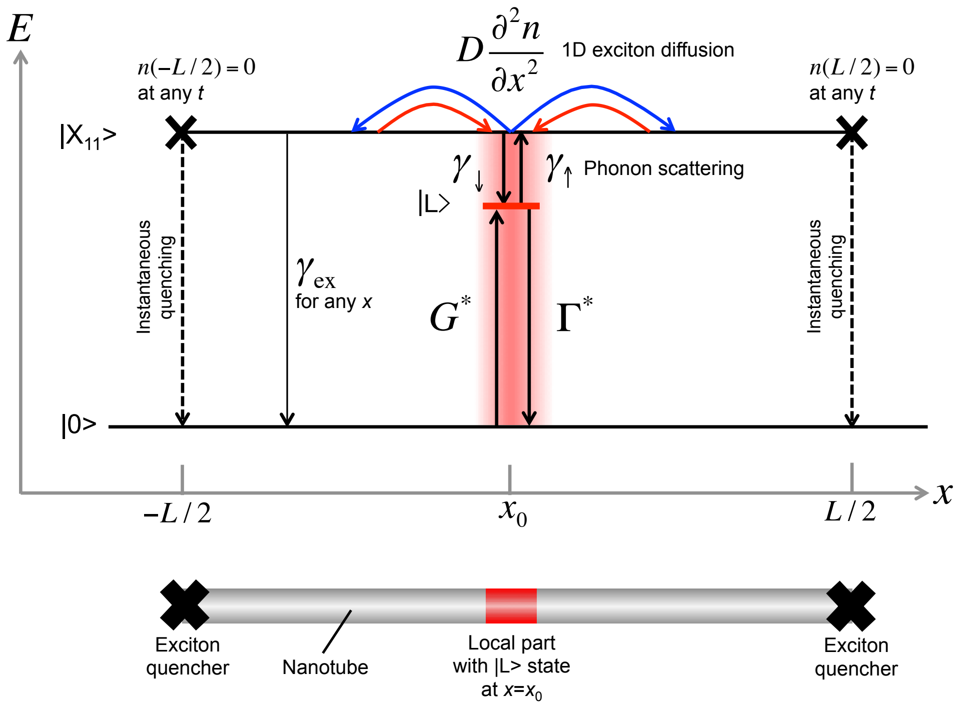

Supplementary Figure 10 | Diagram of the analyzed system. The shaded part corresponds to a local site in a nanotube. The deep localized exciton state appears as |L> around x = x_0. Free X_{11} exciton state with an effective length L (distance between exciton quenching sites) and ground state are shown as |X_{11}> and |0>, respectively. Blue (red) curved arrows indicate the exciton flow out of (into) the local part.

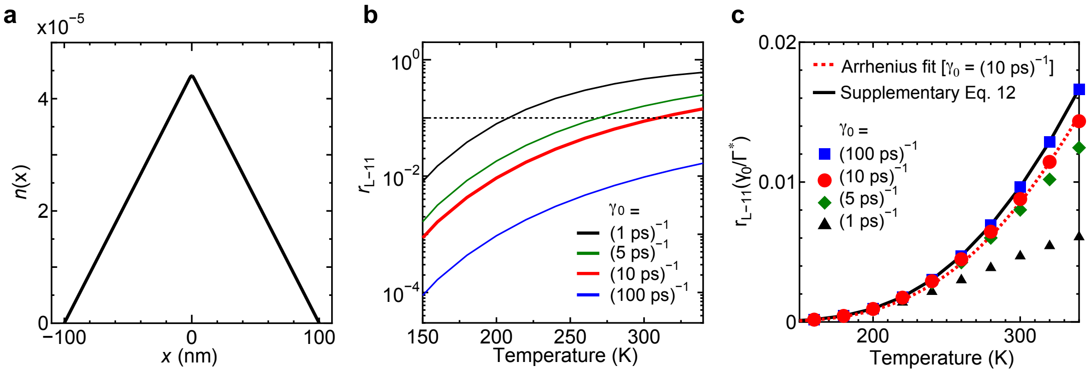

Supplementary Figure 11 | Numerical results for the model system. (**a**) Calculated exciton density $n(x)$ under the steady state condition with $\gamma_0 = (10\text{ ps})^{-1}$, $G^* = (200\text{ ps})^{-1}$, and T = 300 K. (**b**) The net upconversion yield $r_{L\text{-}11}$ calculated using Supplementary Eq. (5) for various γ_0 and T. Horizontal dotted line indicates the experimentally deduced value of $r_{L\text{-}11} \sim 10^{-1}$ for $T \sim 300$ K. (**c**) $r_{L\text{-}11}$ plotted in the unit of γ_0/Γ^* as a function of temperature for various γ_0. Solid curve indicates Supplementary Eq. (12). Red dotted curve is a fit to the data for $\gamma_0 = (10\text{ps})^{-1}$ using Supplementary Eq. (6).

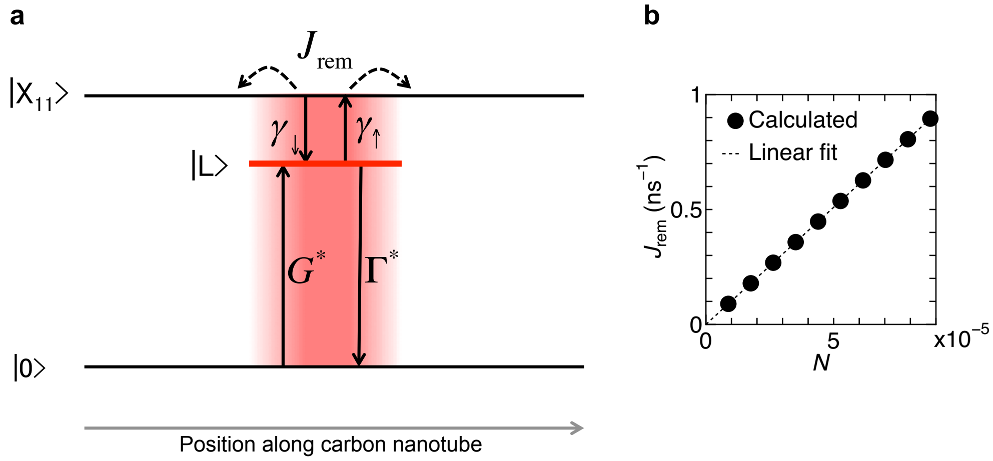

Supplementary Figure 12 | Diagram for the simplified model. (**a**) Schematic of the simplified model of Supplementary Eqs. (7) and (8). Dashed curved arrows indicate the net exciton flux out of the local part J_{rem}, which corresponds to the difference of exciton numbers flowing out of (blue curved arrows in Supplementary Fig. 10) and into (red curved arrows in Supplementary Fig. 10) the local part per unit time. (**b**) Numerical results of the net exciton flux J_{rem} plotted as a function of the effective X_{11} exciton number N in the local part. Dotted line indicates a linear dependence of J_{rem} on N.

Supplementary Notes

Supplementary Note 1: Optical absorption spectra of studied carbon nanotubes

Supplementary Figure 1 shows optical absorption spectra of carbon nanotube ensembles used in this study. Distinct absorption peaks detected at ca. 1.26 eV indicate the dominance of (6, 5) nanotubes in the samples. The small changes in the absorption spectra after the doping treatments indicate that most parts of the nanotubes except for the doped sites remain unchanged, which indicates that the number of doped localized sites are quite small (estimated to be less than one per nanotube) [2,3] and most parts of the nanotubes remain unchanged. We have previously reported that no meaningful increase of optical absorption can be observed around the resonance energy of the localized excitons in the low density doping conditions [2].

Supplementary Note 2: Excitation power density dependence of the upconversion photoluminescence intensity of 0D-doped carbon nanotubes

Supplementary Figure 4 shows the upconversion photoluminescence spectra and corresponding spectral-integrated intensities of as-dispersed (black circles) and 0D-doped (colored circles) nanotubes as functions of excitation power density. Dotted lines in Supplementary Figs. 4b and d represent linear functions obtained under weak excitation power density conditions for comparison. The upconversion photoluminescence intensity exhibited a stronger nonlinear behavior for 0D-doped nanotubes than their as-dispersed counterparts. Artificially introduced 0D-like local states are optically active and exhibit relatively long exciton lifetimes of 70 to 100 ps [2,3]. On the other hand, unintentionally generated states may have shorter lifetimes in as-dispersed nanotubes, as suggested by the absence of clear photoluminescence peaks from these states. Therefore, the unintentionally doped states in as-dispersed nanotubes may present a smaller average exciton population than the artificially introduced optically-active 0D-like local states, possibly resulting in a dissimilar degree of state filling and thus, different nonlinear behaviors.

Supplementary Note 3: Comparison of light penetration depths into highly scattering media for upconversion and visible excitation conditions

Here we demonstrate that the penetration depth of the incident light into highly

scattering media can be extended for near-infrared upconversion excitation conditions compared to normal visible excitation conditions. Supplementary Figure 5 shows (a) a normal optical and (b,c) photoluminescence images of carbon nanotubes dispersed in an opaque medium (0.1% Intralipid® solution [1], Sigma-Aldrich) under upconversion (1.17 eV) and normal visible excitation (2.33 eV) conditions. As shown in Supplementary Fig. 5a, this solution is opaque in the visible optical image. Here, continuous wave solid state lasers were used as the excitation sources, and photoluminescence images in the 1.24–1.31 eV range were selectively captured through optical filters. The normal and upconversion photoluminescence images shown in Supplementary Figs. 5b and 5c indicate that the penetration depth of the incident beam was clearly much longer under upconversion excitation (1.17 eV) than normal visible excitation (2.33 eV) conditions.

Supplementary Note 4: Comparison of normal and upconversion photoluminescence images

Supplementary Figure 6a shows optical image of carbon nanotube dispersion sealed in transparent silicone elastomer held in a glass dish (For details of this sample, please see Supplementary Fig. 7). Here we compare photoluminescence images of the nanotube dispersion under normal (Supplementary Fig. 6b, 2.33 eV) and upconversion (Supplementary Fig. 6c, 1.17 eV) excitation conditions. Continuous wave solid state lasers were used as the excitation sources, and photoluminescence images in the 1.24–1.31 eV range were selectively captured through optical filters. Under the normal excitation condition (Supplementary Fig. 6b), the quality of photoluminescence image was considerably reduced because of unwanted light emission from other materials placed behind the nanotubes. In contrast, under the upconversion excitation condition (Supplementary Fig. 6c), only the photoluminescence of the nanotube dispersion was clearly observed owing to lack of photoemission from any other materials than nanotubes.

Supplementary Note 5: Upconversion photoluminescence imaging of carbon nanotubes behind a mock tissue

Here we demonstrate that carbon nanotubes produce a clear upconversion photoluminescence image behind a mock tissue phantom at a depth reaching 6 mm, under relatively weak upconversion excitation conditions (approximate excitation

power density = 6 mWcm^{-2}), using a Si-based image sensor and a low-cost continuous wave near-infrared laser (1.17 eV). Note that this excitation density (6 mWcm^{-2}) is much smaller than the conservative limit for human skin exposure to near-infrared light at a wavelength of about 1000 nm (ca. 700 mWcm^{-2})[4]. Supplementary Figure 7a shows an optical image of carbon nanotube dispersion sealed in transparent silicone elastomer (SILGARD(R) 184, Sigma-Aldrich) held in a glass dish. Supplementary Figure 7b shows the optical image of the same sample covered with a mock tissue with thickness of 2 mm. 1% Intralipid® solution [1] (Sigma-Aldrich) was used as the mock tissue covering the carbon nanotube dispersion to study the dependence of image clarity on the thickness (*d*) of the tissue. A 1% Intralipid® solution has previously been used in deep-tissue imaging studies using normal near-infrared photoluminescence of carbon nanotubes because of its biological tissue-like scattering properties [1,5]. Supplementary Figure 7c shows a schematic representation of the sample cross-section.

Supplementary Figure 8 shows upconversion photoluminescence images for *d* values from 0 (without mock tissue) to 6 mm. Owing to the negligible autofluorescence or any other photoemission (such as Raman scattering) from other materials than nanotubes, only the photoluminescence from the nanotube dispersion was clearly observed in the upconversion photoluminescence images. The photoluminescence image remained clear even under the mock tissue with thicknesses below 3 mm. For *d* values more than 5 mm, the images lost their sharpness and contrast to some extent but the position of carbon nanotubes could be clearly determined owing to negligible autofluorescence and the long penetration depth of the near-infrared excitation and emission involved in the upconversion imaging.

Supplementary Discussion

Evaluation of the upconversion efficiency

This supplementary section addresses the efficiency of the upconversion processes of excitons trapped in the 0D-like localized states in carbon nanotubes. The ratio between normal and upconversion photoluminescence intensities I_{UCPL}/I_{PL} is expressed as,

$$\frac{I_{UCPL}}{I_{PL}} = \frac{P_L}{P_{22}} \cdot \frac{\sigma_L}{\sigma_{22}} \cdot \frac{N_L}{N_I} \cdot \frac{E_{22}}{E_L} \cdot \frac{r_{L-11}}{r_{22-11}}, \qquad (1)$$

where P_L and P_{22} are the excitation power densities for upconversion and normal photoluminescence at 1.13 (E_L, upconversion excitation photon energy) and 2.18 eV (E_{22}, second subband exciton resonance energy), respectively. σ_{22} and σ_L are the absorption cross sections per carbon atom at E_{22} and E_L, respectively. N_I and N_L correspond to numbers of carbon atoms in the intrinsic 1D part and the intermediate local states for upconversion processes, respectively. $r_{22\text{-}11}$ and $r_{L\text{-}11}$ are net quantum yields for exciton transition from initial excited state (at E_{22} or E_L) to first free exciton state (at E_{11}), respectively.

A comparison between absolute photoluminescence intensities obtained under normal and upconversion excitation conditions gave a ratio $(I_{UCPL}/P_L)/(I_{PL}/P_{22})$ of about 1/235 and 1/140 for as-dispersed and sp^3-doped samples, respectively (Supplementary Fig. 9). Let us define ΔI_{UCPL}, the increased upconversion photoluminescence intensity resulting from additional 0D-like localized states in sp^3-doped samples. Considering Supplementary Eq. (1), $(\Delta I_{UCPL}/P_L)/(I_{PL}/P_{22}) = 1/140 - 1/235 \approx 1/347$ becomes

$$\frac{\Delta I_{UCPL} / P_L}{I_{PL} / P_{22}} = \frac{\sigma_L}{\sigma_{22}} \cdot \frac{\Delta N_L}{N_I} \cdot \frac{E_{22}}{E_L} \cdot \frac{r_{L-11}}{r_{22-11}}, \qquad (2)$$

where ΔN_L is the number of carbon atoms in the additional 0D-like local states. Here, a slight decrease in the normal photoluminescence intensity of free excitons after the doping treatment is neglected. Supplementary Equation (2) provides the rough estimate of the net quantum yield of the exciton upconversion from localized state to free exciton state $r_{L\text{-}11}$. Note that $r_{L\text{-}11}$ obtained using Supplementary Eq. (2) is the upconversion quantum yield for excitons in the additionally (artificially) doped states, and not necessarily equal to that in the initially (unintentionally) doped ones. As will be

demonstrated in the following discussion, we expect that the upconversion quantum yield is approximately proportional to the exciton lifetime in the localized state. From the enhanced nonlinear photoluminescence behavior of 0D-doped nanotubes (Supplementary Fig. 4), we infer that the exciton lifetime in the additional local state is slightly longer than that in the unintentionally doped states (see Supplementary Note 2). The $r_{L\text{-}11}$ for the additional local states thus may be slightly higher than that for the unintentional ones.

Assuming that σ_L is in the same order as that of first subband excitons (σ_{11}), a σ_L/σ_{22} ratio ($\approx \sigma_{11}/\sigma_{22}$) of ca. 2 is obtained using the absorption spectra (Supplementary Fig. 1). The estimated density of the additional localized states in 0D-doped nanotubes is lower than 3 μm^{-1} [2,3]. The small change in optical absorption spectra (Supplementary Fig. 1) and slight reduction in free exciton photoluminescence intensity (Fig. 4 in the main text) after localized state doping are consistent with the low density of the additional localized state. Next, $\Delta N_L/N_I < 0.006$, assuming that the spatial size of the local site is at most as large as that of the free exciton (ca. 2 nm) in (6,5) nanotubes [6]. The reported exciton relaxation quantum yield from state E_{22} to state E_{11}, $r_{22\text{-}11}$, is of the order of 1 [7]. Using these values, the evaluated exciton upconversion quantum yield $r_{L\text{-}11}$ is on the order of 10^{-1} at room temperature for an energy gain of 120 meV. The net upconversion photoluminescence quantum yield obtained by multiplying the normal photoluminescence quantum yield by $r_{L\text{-}11}$ is thus only one order of magnitude smaller than its normal photoluminescence counterpart. Therefore, a net photoluminescence quantum yield of the upconversion photoluminescence is deduced to be about 10^{-3} for an energy gain of 120 meV using a typical normal photoluminescence quantum yield of 10^{-2} for an aqueous (6,5) carbon nanotube suspension [8].

Phenomenological model for the efficient exciton upconversion in a system combining a 1D carbon nanotube and 0D-like local states

Let us now discuss the physical mechanism underlying efficient exciton upconversion phenomena in carbon nanotubes presenting local states. When considering the exciton upconversion from the 0D-like local state into the free exciton state mediated by large energy phonons in carbon nanotubes, it is reasonable to assume that the upconversion rate is proportional to the phonon number n_{ph}. In this case, the

upconverted excitons can be backscattered into the original local state at a rate proportional to $1+n_{ph}$, which is much larger than n_{ph} <0.01 for high-energy optical phonons (E_{ph} ≥ 120 meV) at room temperature. This suggests that efficient phonon-assisted upconversion hardly occurs solely by a simple phonon scattering mechanism. However, for quasi-1D carbon nanotubes with embedded 0D-like local states, upconverted excitons in localized states can be scattered toward spatially distant parts along the 1D nanotube axis through ultrafast exciton scattering processes driving rapid exciton diffusion [9,10] in carbon nanotubes. This 1D exciton diffusion process enables efficient exciton upconversion in carbon nanotubes, as will be discussed below.

We first consider a system shown in Supplementary Fig. 10. For the studied sp^3-doped nanotubes, a deep exciton state below the intrinsic E_{11} state has been predicted as the origin of the local state by density functional theory [11]. Similar local state has also been predicted for oxygen-doped nanotubes [12,13]. In this model, the local part with a deep exciton state |L> is seamlessly connected to the intrinsic part of the 1D carbon nanotube with a length L. Equations for the exciton density $n(x,t)$ in the 1D states |X_{11}> and the exciton population N^* in the local state |L> are written as:

$$\frac{\partial n(x,t)}{\partial t} = D\frac{\partial^2 n(x,t)}{\partial x^2} + [\gamma_\uparrow N^* - \gamma_\downarrow n(x,t)]g(x-x_0) - \gamma_{ex} n(x,t) \qquad (3)$$

$$\frac{dN^*}{dt} = G^* + \gamma_\downarrow \int_{-L/2}^{L/2} n(x,t)g(x-x_0)dx - (\gamma_\uparrow + \Gamma^*)N^* , \qquad (4)$$

where D is a diffusion constant of free excitons in the 1D states, $g(x\text{-}x_0)$ is a function describing the position (x_0) and spatial extent of the local state, γ_{ex} is the intrinsic decay rate of free excitons, G^* is the optical generation function of the localized excitons, Γ^* is the relaxation rate of the localized exciton, and L is the length of the nanotube. In the calculation, we used a normalized Gaussian function $g(x-x_0) = (\sigma\sqrt{2\pi})^{-1}\exp[(x-x_0)^2/2\sigma^2]$ with $x_0 = 0$ and $\sigma = 1$ nm. $\gamma_\uparrow$ and $\gamma_\downarrow$ are phonon-assisted up- and down-conversion rates at the local state expressed as $\gamma_\uparrow = \gamma_0 n_{ph}$ and $\gamma_\downarrow = \gamma_0(1+n_{ph})$, where γ_0 is a rate constant and $n_{ph} = [\exp(E_{ph}/k_B T)-1]^{-1}$ is the corresponding phonon number. Steady state exciton density $n(x)$ and the localized exciton number N^* are obtained by numerically solving Supplementary Eqs. (3) and (4) under conditions $\partial n/\partial t = 0$ and $dN^*/dt = 0$. In this model, we consider diffusion-limited exciton quenching at defects or both ends of a nanotube, which has been believed as the major nonradiative exciton relaxation

pathway in individually dispersed carbon nanotubes in water [8-10,14-16]. In a realistic nanotube with finite number of defect quenching sites, L corresponds to a typical quenching site distance (segment length) in a nanotube. This diffusion-limited exciton quenching effect is taken into account through the boundary conditions $n(\pm L/2) = 0$ [10,14]. The net upconversion quantum yield of the localized exciton $r_{\text{L-11}}$ can be obtained by calculating

$$r_{\text{L-11}} = \frac{\gamma_{\uparrow} N^{*} - \gamma_{\downarrow} \int_{-L/2}^{L/2} n(x) g(x - x_0)\, dx}{G^{*}} \tag{5}$$

for various temperature conditions.

Supplementary Figure 11a shows a solution of $n(x)$, calculated for $\gamma_0 = (10\ \text{ps})^{-1}$, $G^{*} = (200\ \text{ps})^{-1}$, and $T = 300$ K. In all the numerical calculations, we used typical parameters ($D = 5\ \text{cm}^2/\text{s}$, $L = 200$ nm, and $\gamma_{\text{ex}} = (2\ \text{ns})^{-1}$) for individually dispersed micelle-suspended nanotubes in water. We confirmed that variations in these parameters within the reasonable ranges [8,9,15-17] ($D = 1\text{-}20\ \text{cm}^2/\text{s}$, $L = 100\text{-}500$ nm, $\gamma_{\text{ex}} = (1\text{-}5\ \text{ns})^{-1}$) do not affect the conclusion of the following discussion. $\Gamma^{*} = (100\ \text{ps})^{-1}$ was used according to the photoluminescence lifetime of the localized excitons in oxygen-doped nanotubes measured at room temperature [2].

Supplementary Figure 11b shows the calculated upconversion yield $r_{\text{L-11}}$ as a function of temperature T for various γ_0. The experimentally deduced value of the net upconversion yield $r_{\text{L-11}} \sim 10^{-1}$ at $T \sim 300$ K is reproduced for γ_0 on the order of $(10\ \text{ps})^{-1}$. Under this condition, the numerical results of $r_{\text{L-11}}$ below $T \sim 300$ K can be well approximated by an Arrhenius-type behavior (Supplementary Fig. 11c) as:

$$r_{\text{L-11}} = A \exp(-E_{\text{ph}} / k_{\text{B}} T), \tag{6}$$

consistent with the experimental observations of the temperature dependence well fitted by the Arrhenius equation. We found that the upconversion yield nearly scales with $\gamma_0 \Gamma^{*-1}$ and the coefficient A is well approximated as $A \sim \gamma_0 \Gamma^{*-1}$ for $\gamma_0 < \sim (5\ \text{ps})^{-1}$ as shown in Supplementary Fig. 11c. The above analysis thus indicates that the upconversion yield increases when localized exciton lifetime and/or phonon scattering rate expand, and the ratio γ_0/Γ^{*} is a determining factor of the net upconversion yield of the localized excitons.

For a more intuitive understanding of the exciton upconversion physics, here we consider a simplified model as shown in Supplementary Fig. 12a. In this model, we focus on the exciton dynamics only *within* the local part (shaded region in Supplementary Fig. 12a) explicitly as:

$$\frac{dN}{dt} = \gamma_{\uparrow} N^{*} - \gamma_{\downarrow} N - J_{\mathrm{rem}}(N) \tag{7}$$

$$\frac{dN^{*}}{dt} = G^{*} + \gamma_{\downarrow} N - (\gamma_{\uparrow} + \Gamma^{*}) N^{*} , \tag{8}$$

where N is defined as an effective number of X_{11} excitons in the local part, corresponding to $\int n(x)g(x\text{-}x_0)dx$, and J_{rem} is introduced as an *effective exciton removal rate* from the local part via diffusive exciton migration along the 1D axis. J_{rem} is the difference of exciton numbers flowing out of and into the local part per unit time, thus the ratio J_{rem}/G^{*} corresponds to the net upconversion quantum yield $r_{\mathrm{L\text{-}11}}$. The intrinsic recombination rate of N excitons is neglected because it is considered much slower than other processes. J_{rem} should vary depending on N in general. Under conditions in which J_{rem} is a simple function of N, an analytical expression of the upconversion rate $r_{\mathrm{L\text{-}11}}$ can be obtained, which will be helpful for a more intuitive understanding of the mechanism.

Here we examine the dependence of J_{rem} on N. According to Fick's first law, an exciton flux $J_{\mathrm{ex}}(x)$ under steady state condition is expressed as:

$$J_{\mathrm{ex}} = -D\frac{dn}{dx} . \tag{9}$$

The net exciton flux J_{rem} removed from the local part is evaluated as:

$$J_{\mathrm{rem}} = J_{\mathrm{ex}}(x_0 + l) - J_{\mathrm{ex}}(x_0 - l) , \tag{10}$$

where $2l$ is defined as an effective width of the local part and we set $l = 3\sigma$ in the following analysis (variations in $l \geq \sigma$ do not affect the conclusion of the following discussion).

Using Supplementary Eqs. (9) and (10), we numerically calculated J_{rem} and N [$= \int n(x)g(x\text{-}x_0)dx$] by solving Supplementary Eqs. (3) and (4) in the weak excitation conditions $G^{*} \leq \Gamma^{*}$ at T = 300 K. As shown in Supplementary Fig. 12b, the results clearly indicate that J_{rem} is proportional to N, and can be well approximated as $J_{\mathrm{rem}} = \Gamma_{\mathrm{rem}} N$ with a proportionality constant $\Gamma_{\mathrm{rem}} \sim (0.1\ \mathrm{ps})^{-1}$.

Under the steady state condition, the analytical expression of the upconversion yield $r_{\text{L-11}}$ is thus obtained from Supplementary Eqs. (7) and (8) with substitution of $\Gamma_{\text{rem}}N$ for J_{rem} as:

$$r_{\text{L}-11} = \frac{1}{1-\Gamma^{*}/\gamma_{0}+(\Gamma^{*}/\gamma_{0}+\Gamma^{*}/\Gamma_{\text{rem}})\exp(E_{\text{ph}}/k_{\text{B}}T)}. \quad (11)$$

Here, $\Gamma^{*}/\Gamma_{\text{rem}}$ is evaluated to be ~10^{-3} from Γ_{rem} ~ $(0.1\ \text{ps})^{-1}$ and $\Gamma^{*} = (100\ \text{ps})^{-1}$. The phonon scattering rate constant γ_0 has been deduced to be ~ $(10\ \text{ps})^{-1}$, thus Γ^{*}/γ_0 is on the order of 10^{-1}. The phonon energy E_{ph} much exceeds $k_{\text{B}}T$ and $E_{\text{ph}}/k_{\text{B}}T \geq 5$ below $T = 300$ K, indicating $\exp(E_{\text{ph}}/k_{\text{B}}T) > 10^{2}$. Under these conditions, Supplementary Eq. (11) can be well approximated by a simple Arrhenius-type behavior with a prefactor $\gamma_0\Gamma^{*-1}$ as:

$$r_{\text{L}-11} \sim \frac{\gamma_0}{\Gamma^{*}}\exp(-E_{\text{ph}}/k_{\text{B}}T), \quad (12)$$

consistent with the numerical results of Supplementary Eq. (5) as shown in Supplementary Fig. 11c. The simplified model thus provides clear physical insight on the Arrhenius-type temperature dependence and the scaling of $r_{\text{L-11}}$ with $\gamma_0\Gamma^{*-1}$ found for the numerical results of Supplementary Eq. (5).

Supplementary References

1. Welsher, K., Sherlock, S. P. & Dai, H. Deep-tissue anatomical imaging of mice using carbon nanotube fluorophores in the second near-infrared window. *Proc. Natl. Acad. Sci. USA* **108**, 8943-8948 (2011).
2. Miyauchi, Y., Iwamura, M., Mouri, S., Kawazoe, T., Ohtsu, M. & Matsuda, K. Brightening of excitons in carbon nanotubes on dimensionality modification. *Nat. Photonics* **7**, 715-719 (2013).
3. Iwamura, M., Akizuki, N., Miyauchi, Y., Mouri, S., Shaver, J., Gao, Z., Cognet, L., Lounis, B. & Matsuda, K. Nonlinear photoluminescence spectroscopy of carbon nanotubes with localized exciton states. *ACS Nano* **8**, 11254-11260 (2014).
4. Idris, N. M., Gnanasammandhan, M. K., Zhang, J., Ho, P. C., Mahendran, R. & Zhang, Y. In vivo photodynamic therapy using upconversion nanoparticles as remote-controlled nanotransducers. *Nat. Med.* **18**, 1580-1585 (2012).
5. van Staveren, H. J., Moes, C. J. M., van Marie, J., Prahl, S. A. & van Gemert, M. J. C. Light scattering in Intralipid-10% in the wavelength range of 400-1100 nm. *Appl. Opt.* **30**, 4507-4514 (1991).
6. Lüer, L., Hoseinkhani, S., Polli, D., Crochet, J., Hertel, T. & Lanzani, G. Size and mobility of

excitons in (6, 5) carbon nanotubes. *Nat. Phys.* **5**, 54-58 (2009).

7. Lebedkin, S., Hennrich, F., Kiowski, O. & Kappes, M. M. Photophysics of carbon nanotubes in organic polymer-toluene dispersions: Emission and excitation satellites and relaxation pathways. *Phys. Rev. B* **77**, 165429 (2008).

8. Hertel, T., Himmelein, S., Ackermann, T., Stich, D. & Crochet, J. Diffusion limited photoluminescence quantum yields in 1-D semiconductors: Single-wall carbon nanotubes. *ACS Nano* **4**, 7161-7168 (2010).

9. Cognet, L., Tsyboulski, D. A., Rocha, J.-D. R., Doyle, C. D., Tour, J. M. & Weisman, R. B. Stepwise quenching of exciton fluorescence in carbon nanotubes by single-molecule reactions. *Science* **316**, 1465-1468 (2007).

10. Rajan, A., Strano, M. S., Heller, D. A., Hertel, T. & Schulten, K. Length-dependent optical effects in single walled carbon nanotubes. *J. Phys. Chem. B* **112**, 6211-6213 (2008).

11. Piao, Y., Meany, B., Powell, L. R., Valley, N., Kwon, H., Schatz, G. C. & Wang, Y. Brightening of carbon nanotube photoluminescence through the incorporation of sp^3 defects. *Nat. Chem.* **5**, 840-845 (2013).

12. Ghosh, S., Bachilo, S. M., Simonette, R. A., Beckingham, K. M. & Weisman, R. B. Oxygen doping modifies near-infrared band gaps in fluorescent single-walled carbon nanotubes. *Science* **330**, 1656-1659 (2010).

13. Ma, X., Adamska, L., Yamaguchi, H., Yalcin, S. E., Tretiak, S., Doorn, S. K. & Htoon, H. Electronic structure and chemical nature of oxygen dopant states in carbon nanotubes. *ACS Nano* **8**, 10782-10789 (2014).

14. Moritsubo, S., Murai, T., Shimada, T., Murakami, Y., Chiashi, S., Maruyama, S. & Kato, Y. K. Exciton diffusion in air-suspended single-walled carbon nanotubes. *Phys. Rev. Lett.* **104**, 247402 (2010).

15. Miyauchi, Y., Matsuda, K., Yamamoto, Y., Nakashima, N. & Kanemitsu, Y. Length-dependent photoluminescence lifetimes in single-walled carbon nanotubes. *J. Phys. Chem. C* **114**, 12905-12908 (2010).

16. Harrah, D. M. & Swan, A. K. The role of length and defects on optical quantum efficiency and exciton decay dynamics in single-walled carbon nanotubes. *ACS Nano* **5**, 647-655 (2011).

17. Crochet, J. J., Duque, J. G., Werner, J. H., Lounis, B., Cognet, L. & Doorn, S. K. Disorder limited exciton transport in colloidal single-wall carbon nanotubes. *Nano Lett.* **12**, 5091-5096 (2012).
